# Supplementary material for: Disruption of Plasmodium falciparum kinetochore proteins destabilises the nexus between the centrosome equivalent and the mitotic apparatus
Source: Nat Commun. 2024 Jul 10;15:5794. doi: 10.1038/s41467-024-50167-6 (PMC11237077; doi:10.1038/s41467-024-50167-6)
Supplement: Supplementary file 1 — Supplementary Information [file 41467_2024_50167_MOESM1_ESM.pdf]

SUPPLEMENTARY INFORMATION

Disruption of *Plasmodium falciparum* kinetochore proteins destabilises the nexus between the centrosome equivalent and the mitotic apparatus

Jiahong Li[1,2], Gerald J. Shami[3], Benjamin Liffner[4], Ellie Cho[5], Filip Braet[3], Manoj T. Duraisingh[2], Sabrina Absalon[4], Matthew W.A. Dixon[6,7*], Leann Tilley[1*]

[1]Department of Biochemistry and Pharmacology, Bio21 Molecular Science and Biotechnology Institute, The University of Melbourne, Parkville, VIC 3010, Australia; [2]Department of Immunology and Infectious Diseases, Harvard T. H. Chan School of Public Health, Boston, MA, 02115, USA; [3]School of Medical Sciences (Molecular and Cellular Biomedicine) & Australian Centre for Microscopy and Microanalysis, The University of Sydney, NSW 2006; [4]Department of Pharmacology and Toxicology, Indiana University School of Medicine, Indianapolis, Indiana 46202, USA; [5]Biological Optical Microscopy Platform, The University of Melbourne, Parkville, VIC 3010, Australia; [6]Department of Infectious Diseases, The Peter Doherty Institute, The University of Melbourne, Parkville, VIC 3010, Australia; [7]Walter and Eliza Hall Institute, Parkville, VIC, 3010, Australia.

*These authors contributed equally
For correspondence: ltilley@unimelb.edu.au and matthew.dixon@unimelb.edu.au

This PDF file includes:
Supplementary Tables 1 to 3
Supplementary Figures 1 to 16
Supplementary Reference

Supplementary Tables

Supplementary Table 1. Primers used in this study (restriction sites in bold).

Primer name	Sequence (5'-3')
EcoRI-*Pf*NDC80-HR2-Fw	catatttattaaatcta**GAATT**CGGAAAAGTTGTCAGTTCTTC
KasI-*Pf*NDC80-HR2-Rev	gaaaataccgcatca**GGCGCC**CAACACTTTTTTGAAGGTC
*Pf*NDC80-HA Dicre-Fw	cctttaattgtgtgatataatatgcacatttgaactttc
EcoRI-*Pf*Nuf2 HR2-Fw	catatttattaaatcta**GAATT**CCGGCATTAAATGAACATATTGAAC
KasI-*Pf*Nuf2 HR2-Rev	gaaaataccgcatca**GGCGCC**CCTTTTGTATATTGTTGAGTACCTTG
*Pf*Nuf2-HA Dicre-Fw	gaaaaataaattgtatgaacagccataattctgtatagccaaaaag
HA-Rev	TAGTCCGGGACGTCGTACGG
glmS-Rev	CGAACATTAAGCTGCCATATCCCTCG

Supplementary Table 2. Alt-R CRISPR-Cas9 crRNA sequence used in this study.

Parasite line	Alt-R CRISPR-Cas9 crRNA sequence
*Pf*Nuf2-HA-glmS-Dicre	ATTTCTTCAAAACCAAGTCG
*Pf*NDC80-HA-glmS-Dicre	ATCTGTATATGTAAAAAAGG

Supplementary Table 3. Primary and secondary antibodies used in expansion microscopy.

Primary Antibody	Species	Dilution	Sources
Anti-β tubulin (clone TUB 2.1)	Mouse	1:500	Sigma-Aldrich (T5201)
Anti-HA (c29F4)	Rabbit	1:250	NEB (3724S)
Anti-GFP (4B10)	Mouse	1:200	Cell Signaling Technology (2955S)
Anti-*Pf*GAP45	Rabbit	1:500	Baum *et al* [1]
Anti-centrin (clone 20H5)	Mouse	1:250	Sigma-Aldrich (630249)
Anti-α tubulin	Rabbit	1:200	Sigma (SAB3501072)
Anti-α tubulin	Rabbit	1:100	ABclonal (AC007)
Anti-polyE (pAb IN105)	Rabbit	1:500	AdipoGen Life Sciences (AG-25B-0030-C050)
**Secondary antibodies**	**Species**	**Dilution**	**Sources**
anti-mouse IgG Alexa Fluor 568	Goat	1:500	Invitrogen (A-11004)
anti-rabbit IgG Alexa Fluor 647	Goat	1:500	Invitrogen (A-21245)

Supplementary Figures

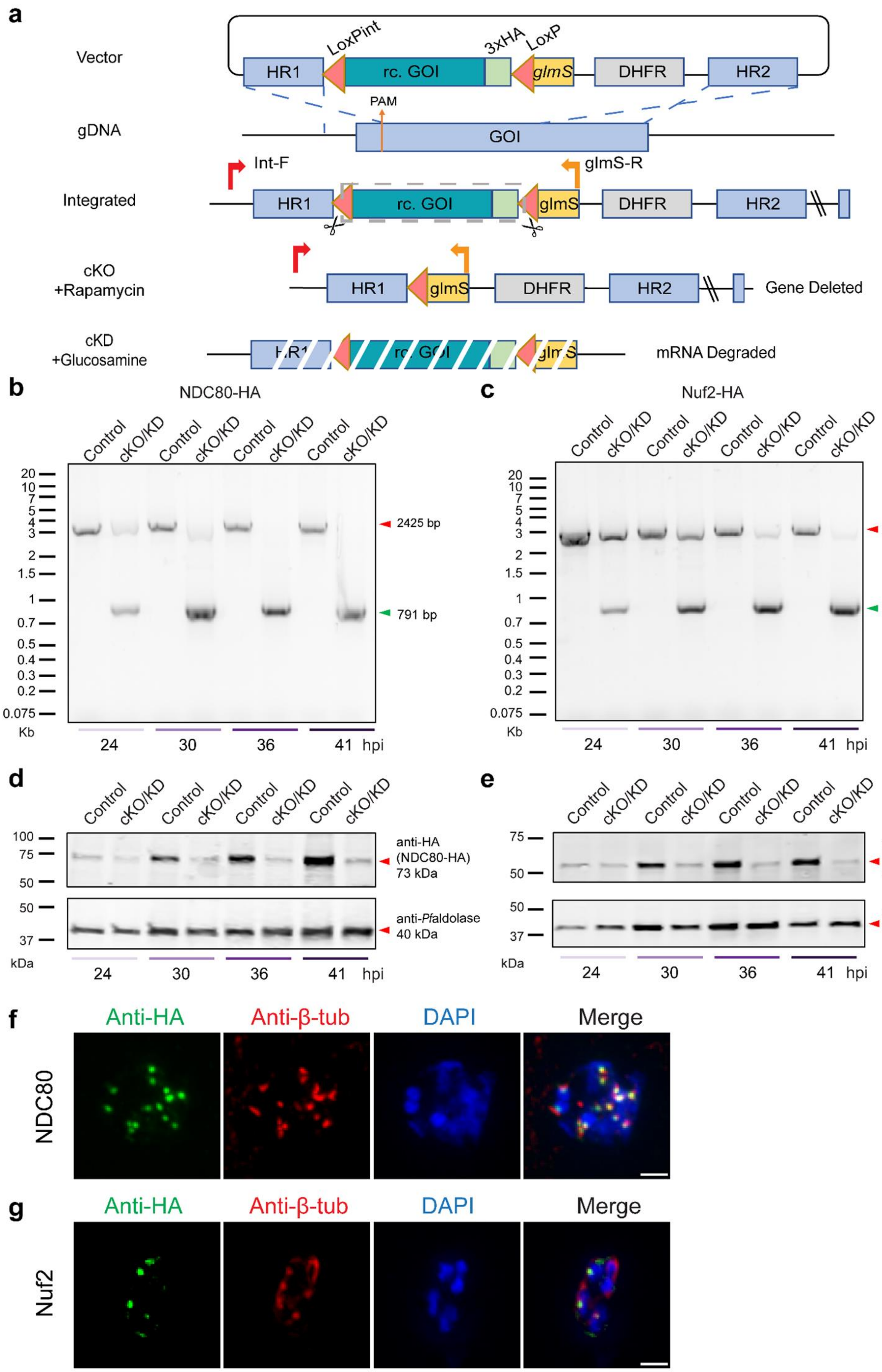

Supplementary Figure 1. Validation of *Pf*NDC80-HA and *Pf*Nuf2-HA parasite lines. a Schematic of the constructs for conditional disruption of *Pf*NDC80 and *Pf*Nuf2 expression by rapamycin-induced gene excision (knockout, KO) and glucosamine-induced mRNA self-cleavage (knockdown, KD). rc denotes the recodonised

ndc80/*nuf2* sequence, GOI is gene of interest (*ndc80*/*nuf2*) and LoxPint is *loxP* artificial introns (coral arrowheads). PAM is protospacer adjacent motif, which is the target site for the CRISPR-Cas9 system to sever the downstream DNA sequence. **b, c** PCR analysis at different time points confirms the correct integration of the plasmid into the *ndc80* (**b**) and *nuf2* (**c**) loci and illustrates the progress of gene deletion. The integration forward (IntF) primer was used with the glmS-R reverse primers to screen for integration and gene excision. The *ndc80-glmS* product is 2425 bp and the excised *ndc80-glmS* product is 791 bp. The *nuf2-glmS* product is 2288 bp and the excised *nuf2-glmS* product is 746 bp. **d, e** *Pf*NDC80-HA (73 kDa) (**d**) and *Pf*Nuf2-HA (58 kDa) (**e**) proteins are detected by Western blotting (anti-HA) at the expected sizes at different time points, illustrating efficient cKO/KD, when compared to DMSO controls. Anti-*Pf*aldolase (40 kDa) is used as a loading control. Samples for PCR and Western blotting were collected at 24, 30, 36 and 41 hpi. The experiment was performed two times. **f, g** Immunofluorescence microscopy of samples labelled with anti-HA, anti-β-tubulin (anti-β-tub) and DAPI, showing the location of *Pf*NDC80 (**f**) and *Pf*Nuf2 (**g**) in mitotic schizonts at 36 hpi. All images are displayed as z-projections. The experiment was performed three times. All scale bars are 2 μm.

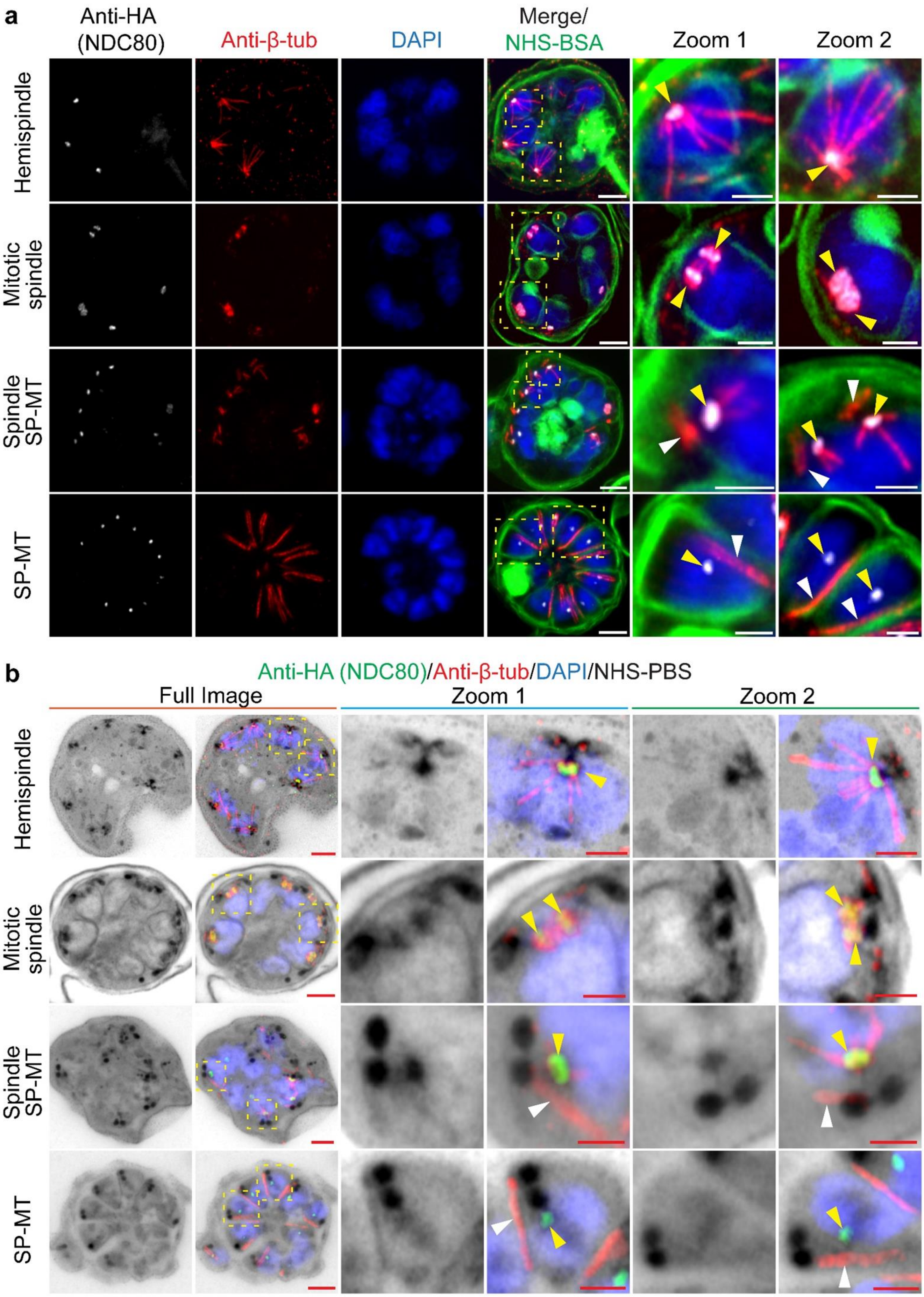

Supplementary Figure 2. Additional examples and individual channels illustrating *Pf*NDC80-HA location in *P. falciparum* schizonts. a, b U-ExM images of *Pf*NDC80-HA transfectants showing *Pf*NDC80-HA location relative to hemispindle, mitotic spindle and subpellicular microtubules. NHS-BSA labelling was

used to highlight membranes (**a,** green) while NHS-PBS staining was used to highlight protein rich structures (**b**, inverse greyscale). *Pf*NDC80-HA was labelled with anti-HA (**a,** greyscale or **b,** green, yellow arrowheads), microtubules with anti-β-tubulin (red, anti-β tub) and chromatin with DAPI (blue). Sub-pellicular microtubules are indicated by white arrows. The data relate to Fig. 1c. All images are displayed as z-projections. The experiment was performed three times. All scale bars are 5 µm, except the zoom images which are 2 µm.

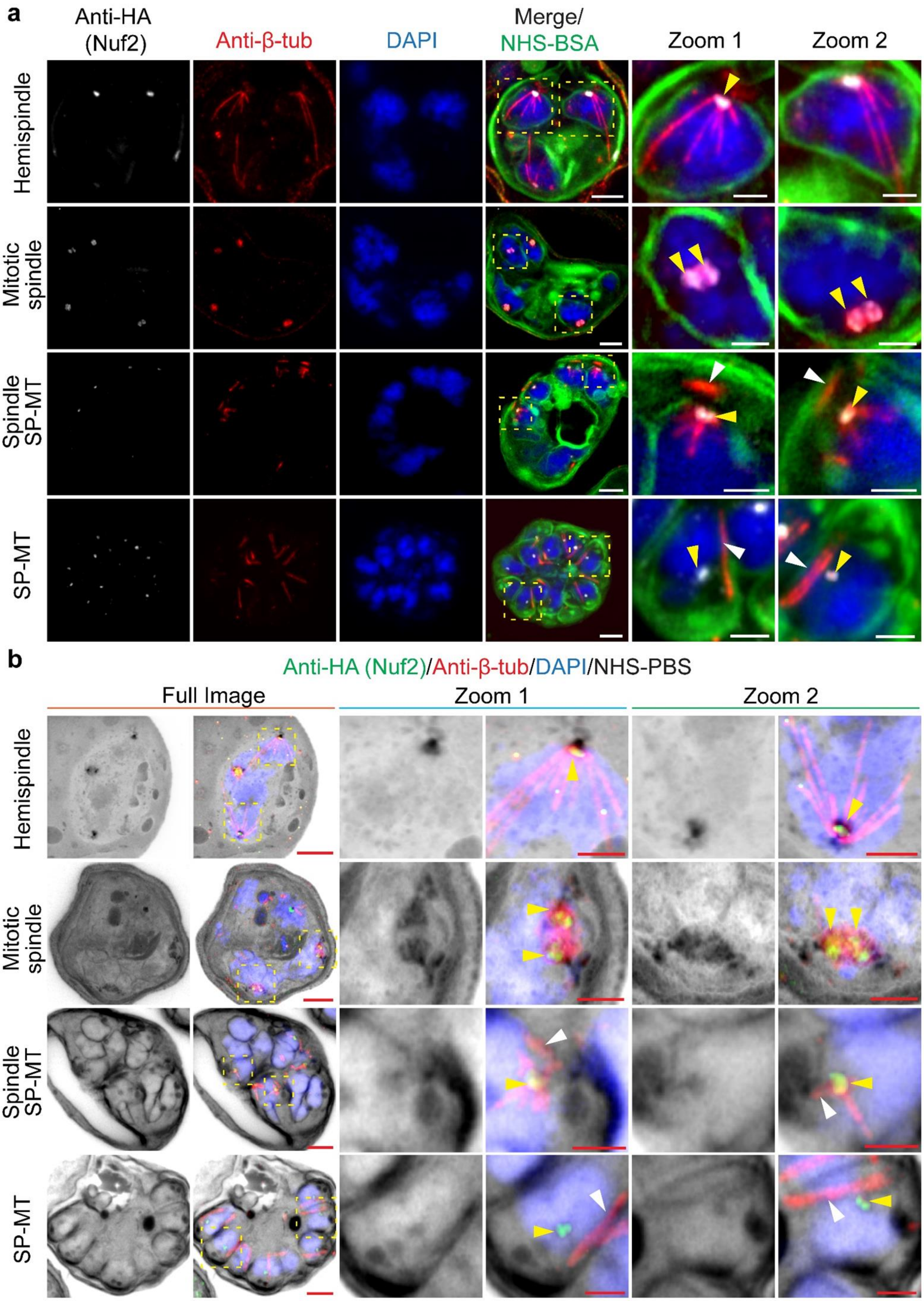

Supplementary Figure 3. Additional examples and individual channels illustrating *Pf*Nuf2-HA location in *P. falciparum* schizonts. a, b U-ExM images of *Pf*Nuf2-HA transfectants showing *Pf*Nuf2-HA location relative to hemispindle, mitotic spindle and subpellicular microtubules, labelled with NHS-BSA (**a,** green) or

NHS-PBS (**b**, inverse greyscale). *Pf*Nuf2-HA was labelled with anti-HA (**a,** greyscale or **b,** green, yellow arrowheads), microtubules with anti-β-tubulin (red, anti-β tub) and chromatin with DAPI (blue). Sub-pellicular microtubules are indicated by white arrows. The data relate to Fig. 1c. All images are displayed as z-projections. The experiment was performed three times. All scale bars are 5 µm, except the zoom images which are 2 µm.

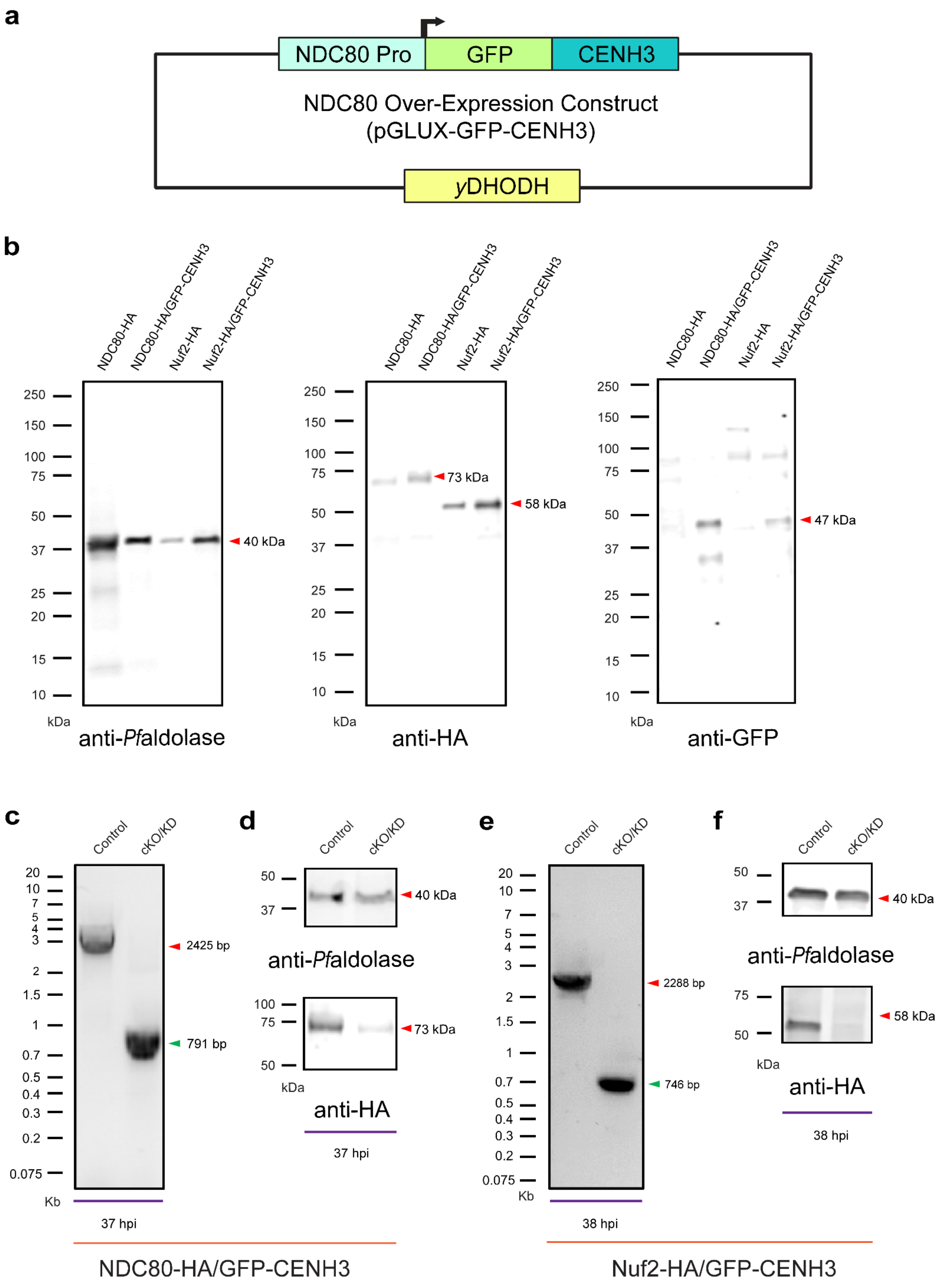

Supplementary Figure 4. Validation of *Pf*NDC80-HA/GFP-*Pf*CENH3 and *Pf*Nuf2-HA/GFP-*Pf*CENH3 parasite lines. a Schematic of the constructs for GFP-*Pf*CENH3 reporter co-transfected into the *Pf*NDC80-

HA and *Pf*Nuf2-HA parasite lines. **b** Western blotting of *Pf*NDC80-HA vs *Pf*NDC80-HA/GFP-*Pf*CENH3 and *Pf*Nuf2-HA vs *Pf*Nuf2-HA/GFP-*Pf*CENH3 parasite lines, detected using anti-HA (*Pf*NDC80-HA, 73 kDa; and *Pf*Nuf2-HA, 58 kDa) and anti-GFP (GFP-*Pf*CENH3, 47 kDa). Anti-*Pf*aldolase was used as a loading control (40 kDa). **c, d** Genomic DNA PCR and Western blotting reveal efficient excision of *ndc80* (**c**) and reduction of *Pf*NDC80-HA (**d**) protein expression. Sample of *Pf*NDC80-HA/GFP-*Pf*CENH3 conditional knockout/knockdown (cKO/KD) schizonts were collected at 37 hpi. **e, f** The *nuf2* gene (**e**) was successfully excised and *Pf*Nuf2-HA protein (**f**) expression was significant reduced in *Pf*Nuf2-HA/GFP-*Pf*CENH3 cKO/KD schizonts (38 hpi). Western blotting and genomic DNA PCR experiments were performed three times. The integration forward (IntF) primer was used with the glmS-R reverse primers to screen for integration and gene excision. For primer locations used for PCR refer to Supplementary Fig. 1a.

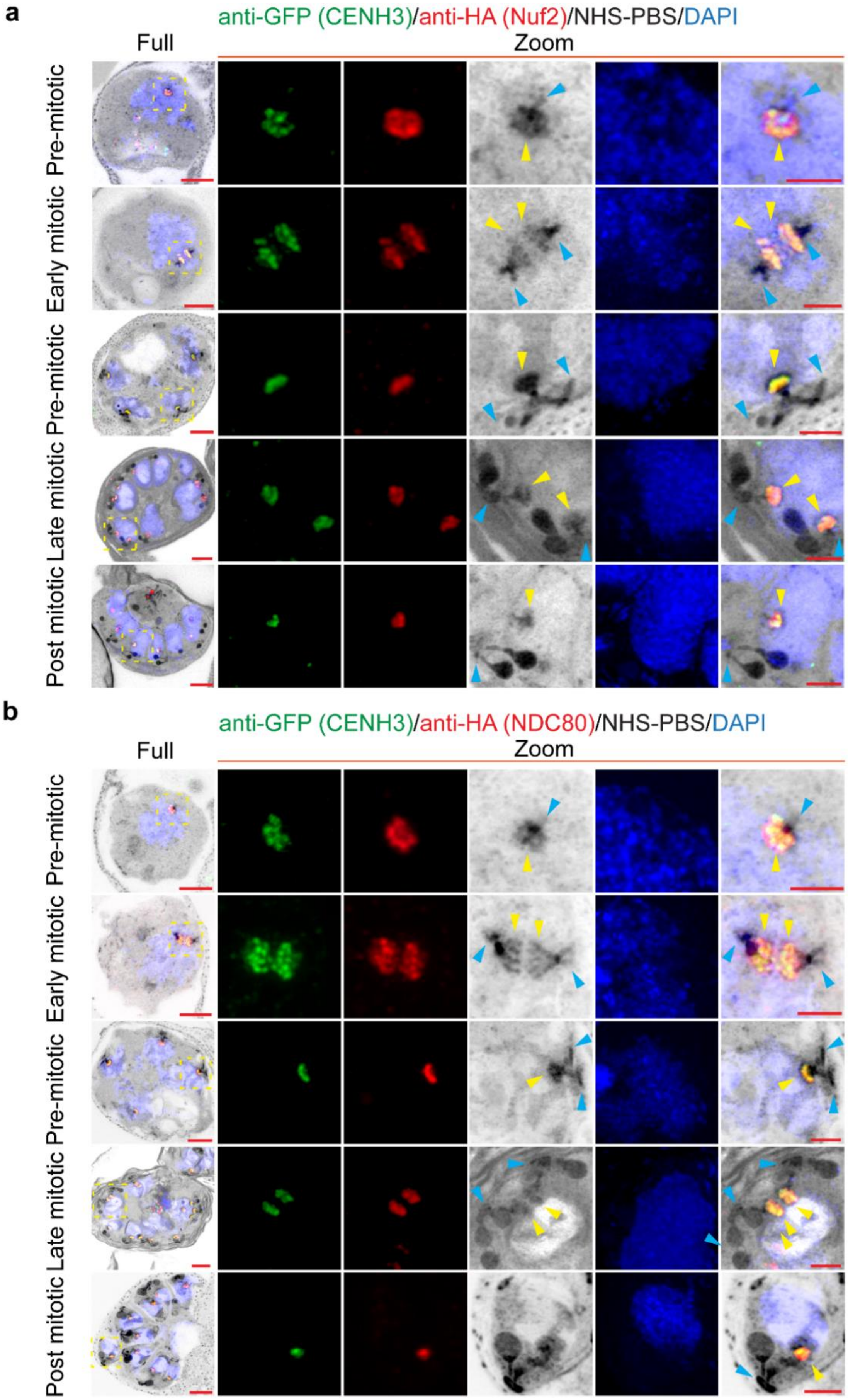

Supplementary Figure 5. Additional examples and individual channels illustrating *Pf*Nuf2-HA/GFP-*Pf*CENH3 and *Pf*NDC80-HA/GFP-*Pf*CENH3 location in *P. falciparum* schizonts. a, b U-ExM images of *Pf*Nuf2-HA/GFP-*Pf*CENH3 (**a**) and *Pf*NDC80-HA/GFP-*Pf*CENH3 (**b**) transfectants showing *Pf*Nuf2-HA or *Pf*NDC80-HA location relative to the centromere (GFP-*Pf*CENH3). The cells are counterstained with NHS-

PBS (inverse greyscale). *Pf*Nuf2-HA or *Pf*NDC80-HA was labelled with anti-HA (green, yellow arrowheads) and *Pf*CENH3 with anti-GFP (red, yellow arrowheads). The signals overlap with the anti-HA signal shown, appearing yellow in the merged images. The chromatin is labelled with DAPI (blue). The outer centriolar plaque structures are indicated by aqua arrowheads. The data relate to Fig. 1d. The experiment was performed three times. All images are displayed as z-projections. All scale bars are 5 µm, except the zoom images which are 2 µm.

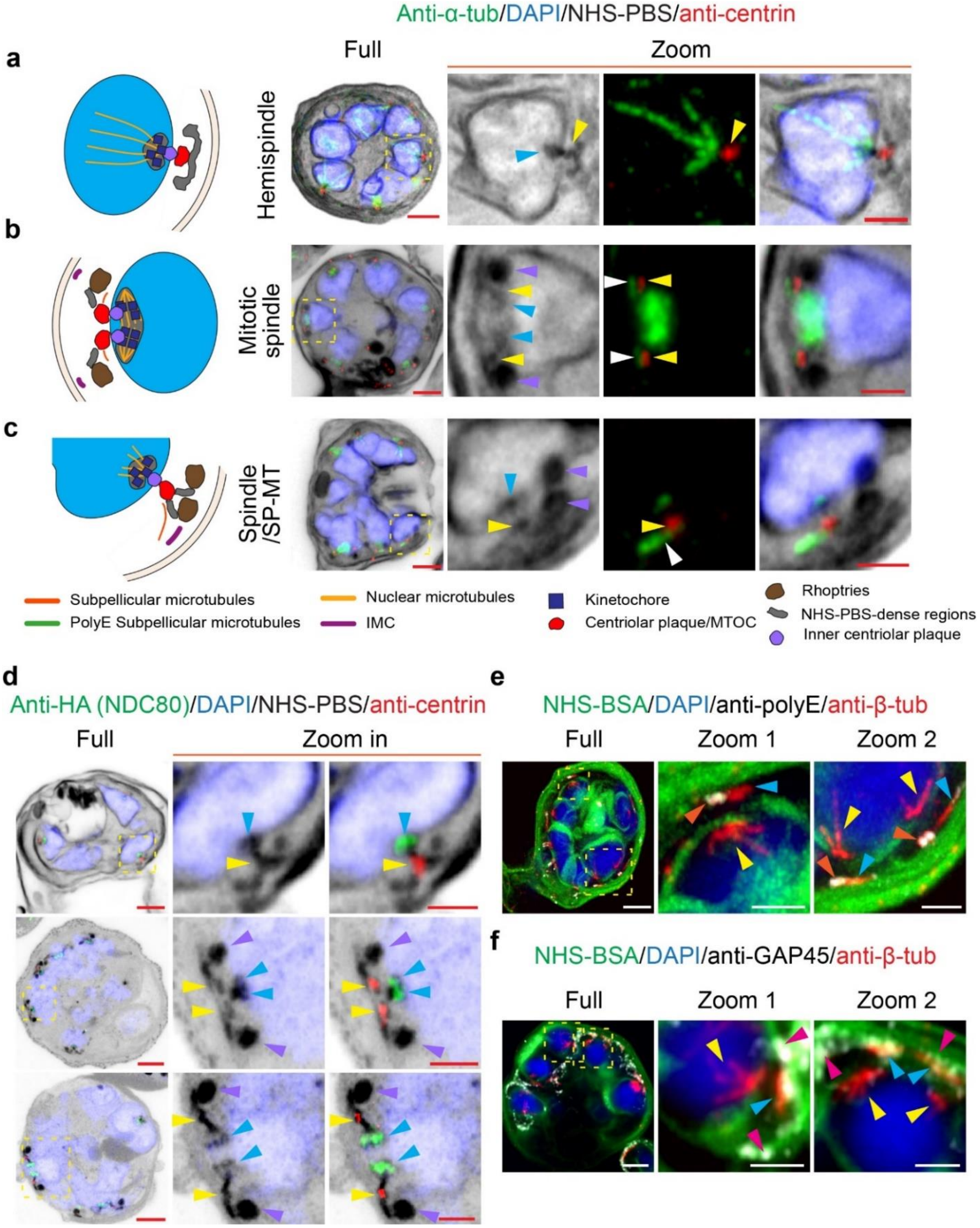

Supplementary Figure 6. Reorganisation of the mitotic machinery and apical organelles during mitotic division. a-c Different stages of nuclear division and schizogony imaged by U-ExM in samples labelled with NHS-PBS (inverse greyscale), anti-α-tubulin (green, anti-α-tub), chromatin (blue) and the outer centriolar plaque marker, anti-centrin (red). An illustration for each stage shown. Microtubules are evident as a hemispindle (**a**), a mitotic spindle (**b**), and a post-mitotic spindle remnant (**c**) and sub-pellicular microtubules (**c**, white arrowheads) as the *Pf*NDC80-HA parasite matures. Centrin is associated with an NHS-PBS-labelled structure just outside the nucleus (yellow arrowheads). NHS-PBS-labelled structures (aqua arrowheads; inner centriolar plaque) are evident at the site of origin of the hemispindles (**a**), associated with the mitotic spindle (**b**) and at the site of the post-mitotic spindle remnant (**c**). NHS-PBS-labelled rhoptries (purple arrowheads)

appear to be closely associated with the outer centriolar plaque. The experiment was performed three times. (**d**) Locations of the kinetochore marker, *Pf*NDC80-HA (green, aqua arrowheads), relative to centrin (red, yellow arrowheads) and NHS-PBS-labelled rhoptries (purple arrowheads). The experiment was performed twice. (**e, f**) U-ExM of NHS-BSA-labelled parasites (green) showing different microtubule populations (anti-β-tubulin, anti-β-tub, red) in late mitotic schizonts. The minus-end of the subpellicular microtubules (aqua arrowheads) is marked with anti-polyE (greyscale, orange arrowheads) (**e**), while the inner membrane complex is marked with anti-anti-*Pf*GAP45 (anti-GAP45, greyscale, magenta arrowheads) (**f**). The spindle remnants are indicated with yellow arrowheads. DAPI stains the chromatin (blue). The images are displayed as z-projections. Supplementary Fig. 6e experiment was performed one time and 6f was performed two times. Full image/zoom scale bars are 5 μm and 2 μm. Additional images are presented in Supplementary Fig. 7-9.

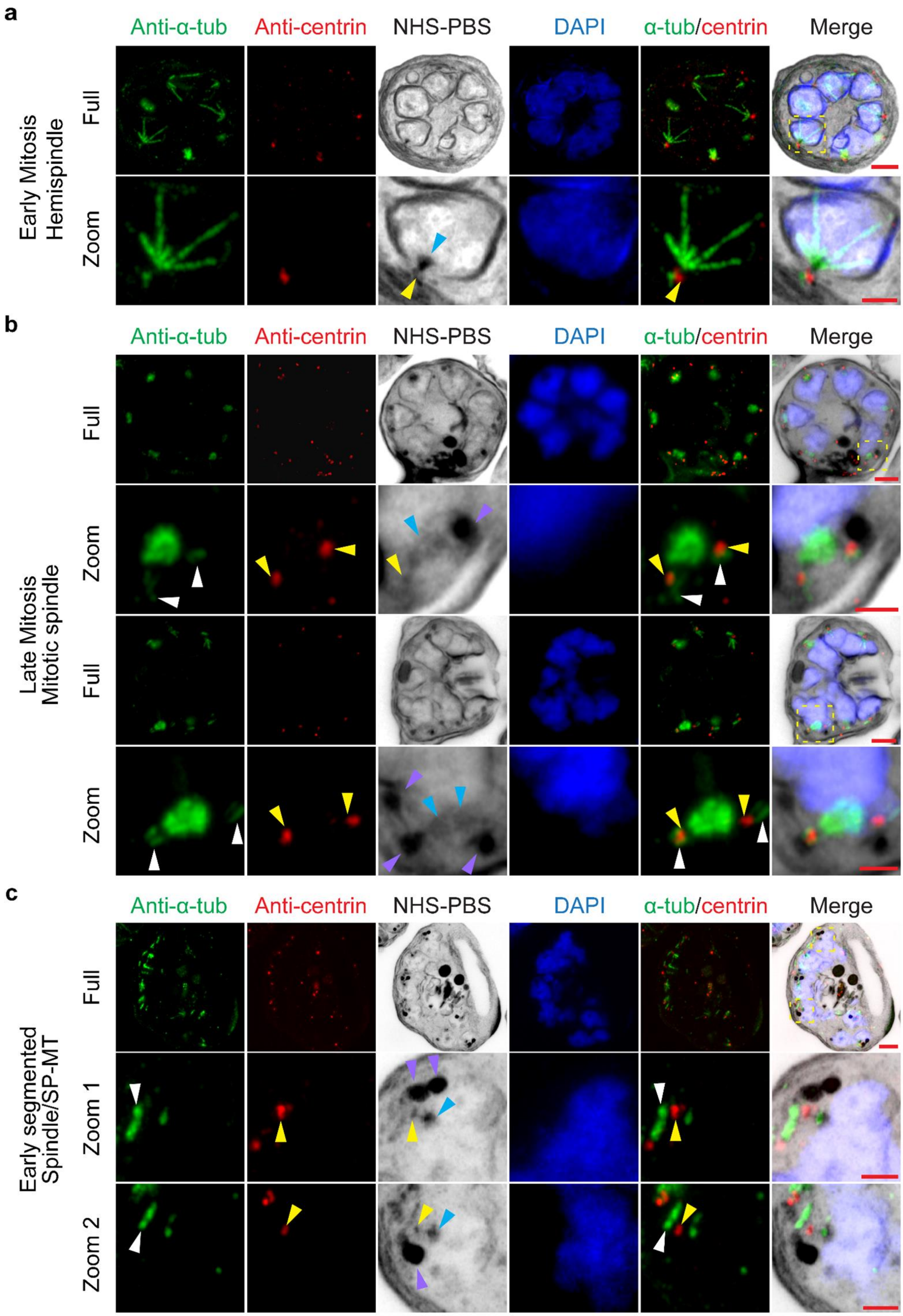

Supplementary Figure 7. Additional examples of *Pf*NDC80-HA and individual channels illustrating tubulin and centrin organisation in schizont stages. U-ExM images of hemispindles (**a**), mitotic spindles (**b**) and spindle remnants and sub-pellicular microtubules (SP-MT) (**c**) in the *Pf*NDC80-HA line. The microtubules are labelled with anti-α-tubulin (green, anti-α-tub), DAPI (blue) and NHS-PBS (inverse

greyscale). The outer centriolar plaque is labelled with anti-centrin (red, yellow arrowheads). The inner centriolar plaque structures are evident as NHS-PBS-labelled puncta (aqua arrowheads). The nascent subpellicular microtubules (white arrowheads) and rhoptries (purple arrowheads) are evident in a later round of nuclear division (b) and in an early segmented schizont (**c**). The data extend Supplementary Fig. 6a-c. All images are displayed as z-projections. The experiment was performed three times. All scale bars are 5 μm, except the zoom images which are 2 μm.

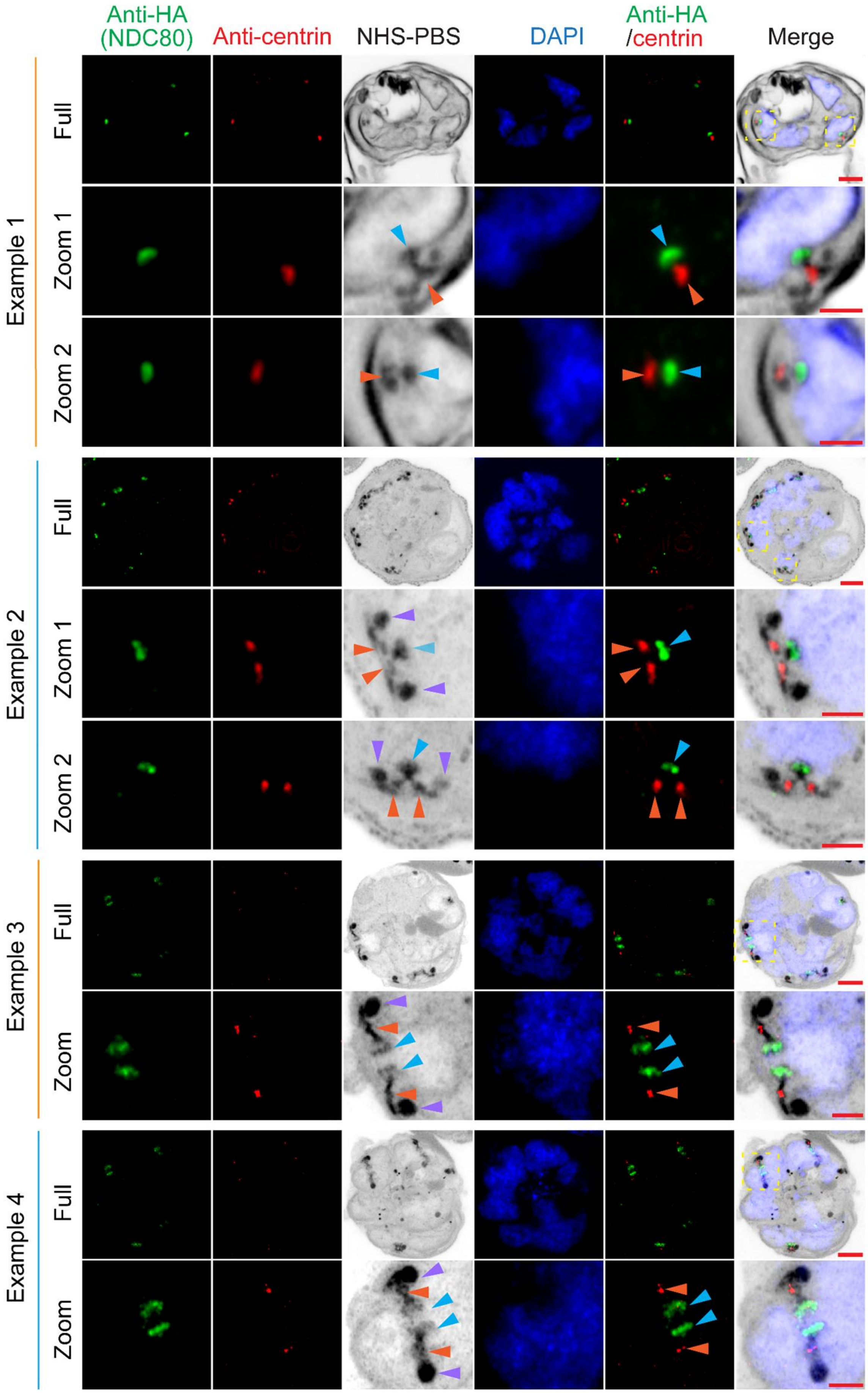

Supplementary Figure 8. Additional examples and individual channels illustrating kinetochore and centrin organisation in schizont stages. U-ExM images of early segmented schizonts (*Pf*NDC80-HA line) showing kinetochores labelled with anti-HA (green, aqua arrowheads), outer centriolar plaque labelled with

anti-centrin (red, orange arrowheads), chromatin marked with DAPI (blue), and NHS-PBS (inverse greyscale). The nascent rhoptries are NHS-PBS-labelled (purple arrowheads). A physical nexus is evident between the kinetochore-containing compartment, at the nuclear periphery, through to the outer centriolar plaque and the apical organelles. These data extend the data in Supplementary Fig. 6d. All images are displayed as z-projections. The experiment was performed twice. All scale bars are 5 μm, except the zoom images which are 2 μm.

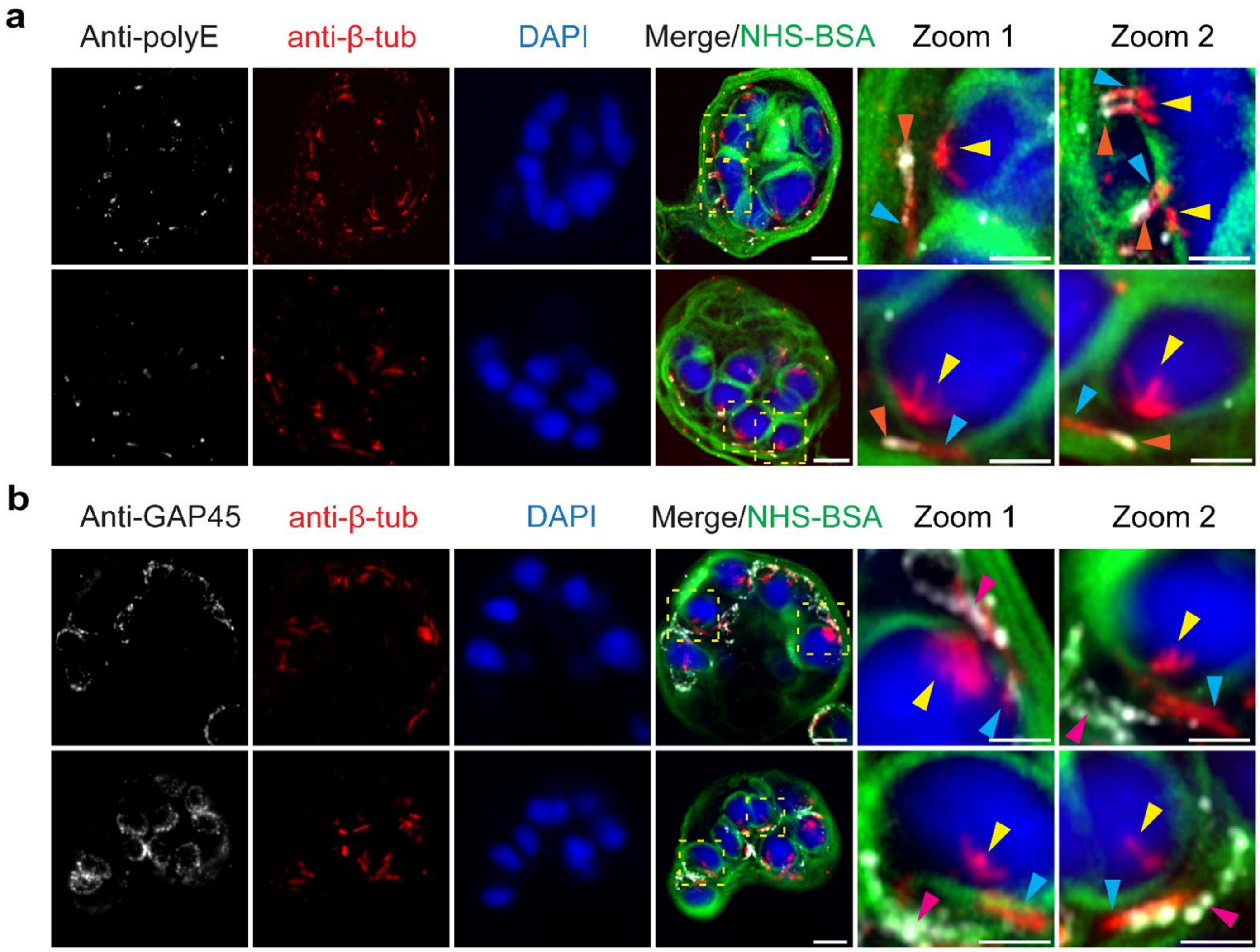

Supplementary Figure 9. Additional examples and individual channels illustrating microtubules, polyglutamylation and IMC organisation during schizont segmentation. U-ExM images of *Pf*NDC80-HA transfectants showing the location of microtubules (**a, b,** anti-β-tubulin, anti-β-tub, red), polyglutamylation modification (**a**, anti-polyE, greyscale, orange arrowheads) and an inner membrane complex marker (**b,** anti-*Pf*GAP45, anti-GAP45, greyscale, magenta arrowheads). Chromatin was labelled with DAPI (blue). The general protein label was NHS-BSA (green). These data extend the data in Supplementary Fig. 6e, f. Supplementary Fig. 9a experiment was performed one time and 9b was performed two times. All images are displayed as z-projections. All scale bars are 5 μm, except the zoom-in images, which are 2 μm.

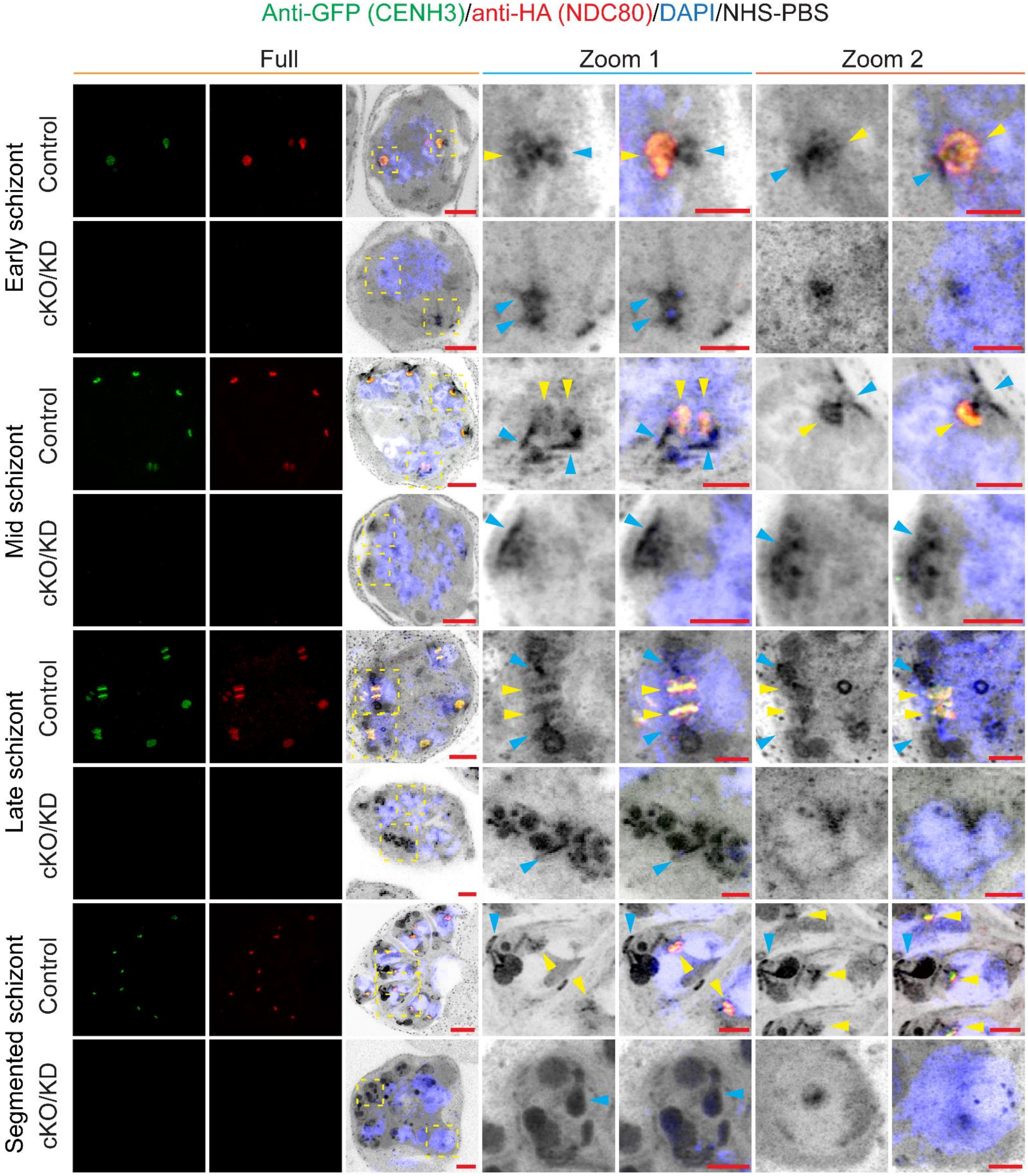

Supplementary Figure 10. Additional examples of the effect of *Pf*NDC80-HA/GFP-*Pf*CENH3 conditional knockout/knockdown on centromere organisation. U-ExM images of control (DMSO) and *Pf*NDC80-cKO/KD (conditional knockout/knockdown, treated with rapamycin and glucosamine) in early, mid, late and mature stage schizonts. NHS-PBS (inverse greyscale) is the general protein label and DAPI (blue) stains the chromatin. In the merge channel of control cells, the signal for the centromere protein, *Pf*CENH3 (CENH3; anti-GFP, green) overlaps with the signal for *Pf*NDC80-HA (anti-HA, red), as indicated with yellow arrowheads. Both the anti-HA and anti-GFP signals are lost in cKO/KD cells. The outer centriolar plaque is indicated with aqua arrowheads. The data relate to Fig. 3a. All images are displayed as z-projections. The experiment was performed three times. All scale bars are 5 µm, except the zoom images which are 2 µm.

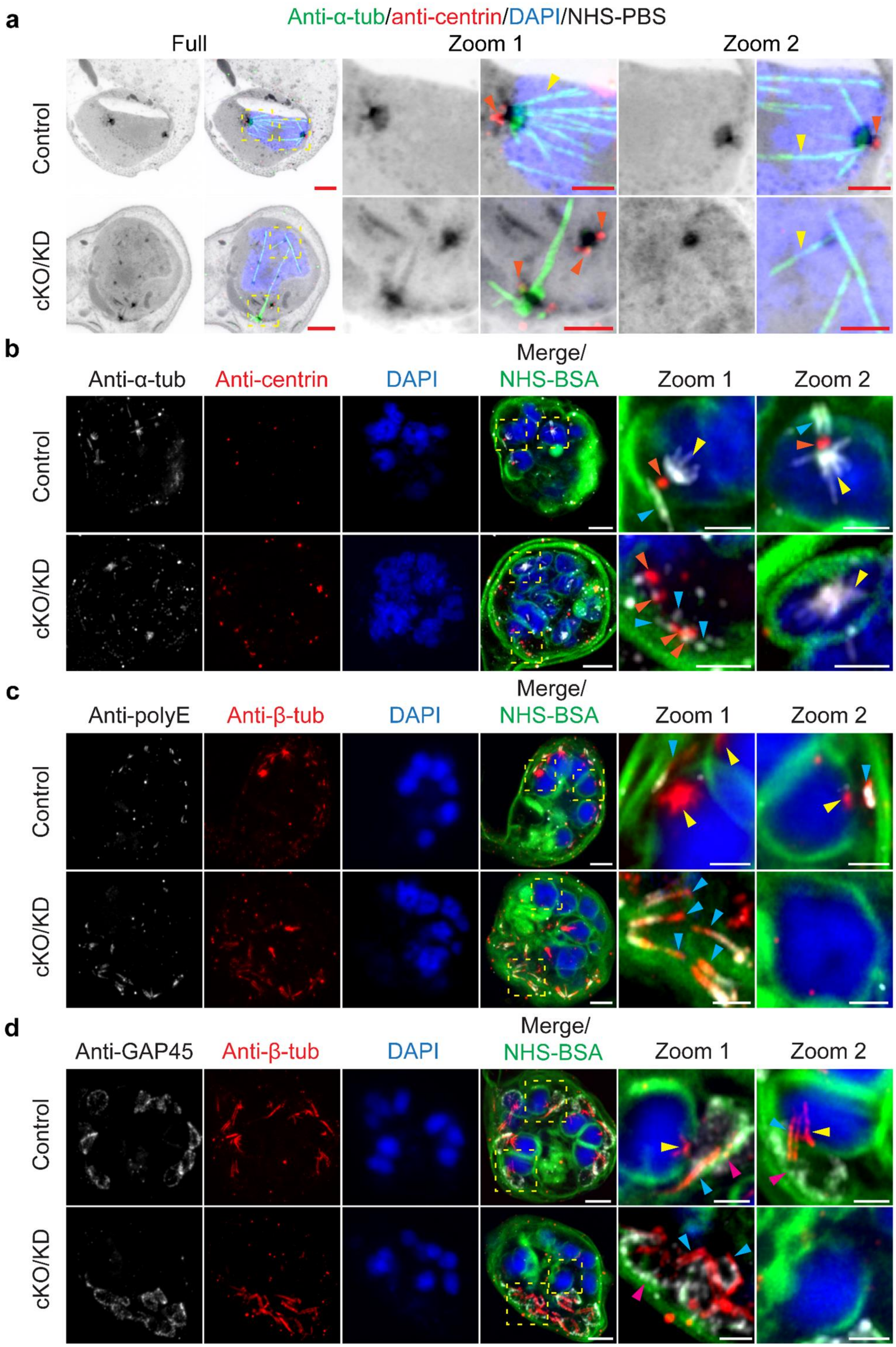

Supplementary Figure 11. Additional examples of the effect of *Pf*NDC80-HA conditional

knockout/knockdown on nuclear division. U-ExM images of control (DMSO) and *Pf*NDC80-cKO/KD (conditional knockout/knockdown, treated with rapamycin and glucosamine) in early stage mitotic schizonts (**a**), late mitotic schizonts (**b)** and early segmented schizonts (**c, d**). NHS-PBS (inverse greyscale) or NHS-BSA (green) are the general protein labels and DAPI (blue) stains the chromatin. Anti-α-tubulin (green or greyscale, anti-α-tub) or anti-β-tubulin (red, anti-β-tub) are used to label microtubules. Spindle microtubules (**a**) and remnant spindle microtubules (**b, c, d**) are indicated with yellow arrowheads. Sub-pellicular microtubules are indicated with aqua arrowheads. **a, b.** Anti-centrin (red, orange arrowheads) labels the outer centriolar plaque. The data relate to Fig. 3c. The experiment was performed twice. **c.** Anti-polyE (greyscale) indicates sub-pellicular microtubules (aqua arrowheads), which are also labelled with anti-β-tubulin (red). The data relate to Fig. 3e. The experiment was performed one time. **d.** Anti-*Pf*GAP45 (greyscale) labels the inner membrane complex (magenta arrowheads). The data relate to Fig. 3f. The experiment was performed twice. All images are displayed as z-projections. All scale bars are 5 μm, except the zoom images, which are 2 μm.

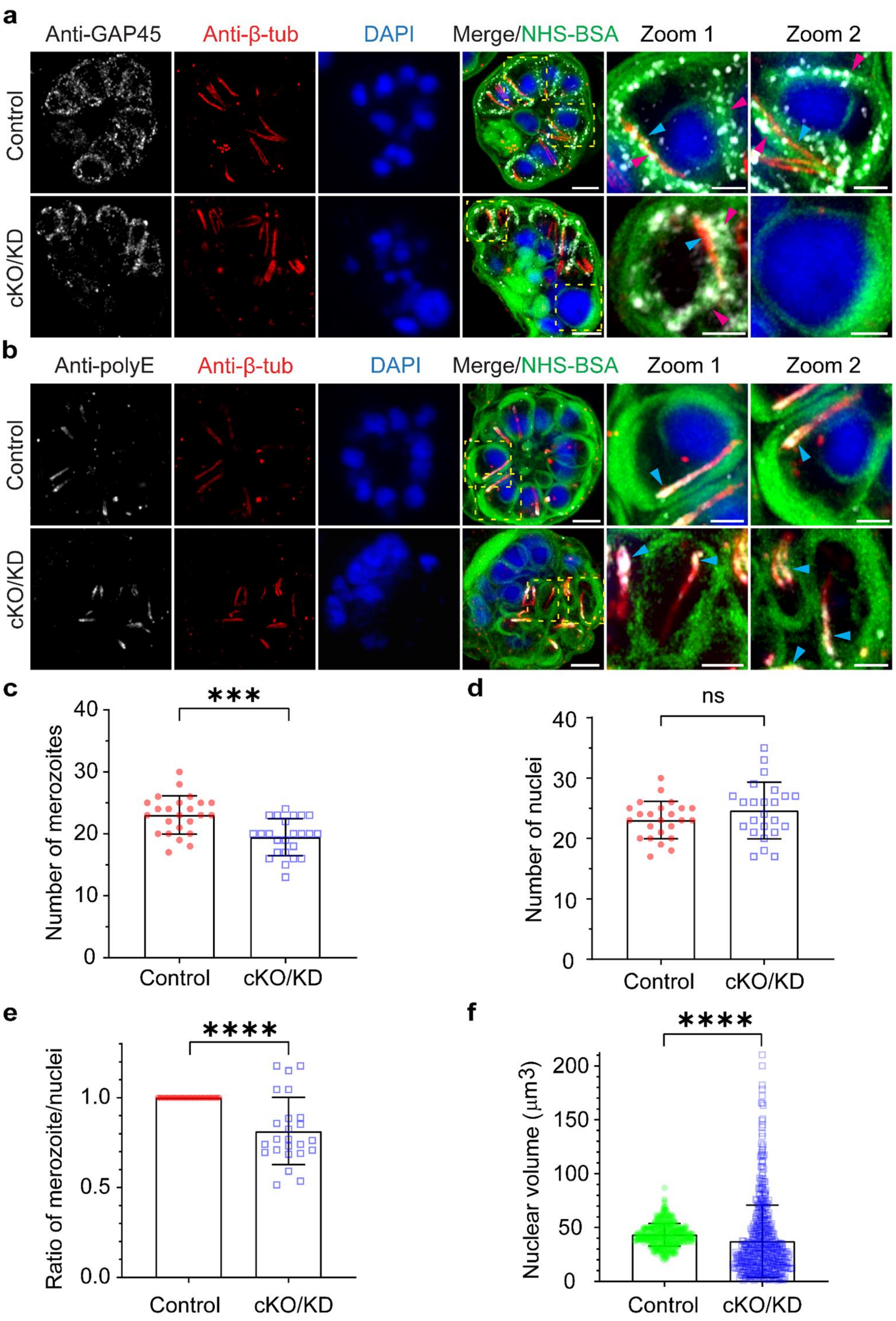

Supplementary Figure 12. Additional examples of the effect of *Pf*NDC80 conditional knockout/knockdown on cytokinesis. U-ExM images of NHS-BSA-labelled (green) *Pf*NDC80-HA transfectants showing subpellicular microtubules (**a, b,** red, anti-β-tubulin (anti-β-tub), aqua arrowheads), inner membrane complex (**a,** greyscale, anti-*Pf*GAP45 (anti-GAP45), magenta arrowheads) and polyglutamylation modification (**b**, greyscale, anti-polyE) in fully segmented schizonts (41 hpi). DAPI (blue)

stains the chromatin. The data relate to Fig. 4a, b. Supplementary Fig. 12a experiments were performed twice and 12b one time. All images are displayed as z-projections. All scale bars are 5 μm, except the zoom-in images which are 2 μm. Quantification of nuclei (**c**) and merozoite (**d**) numbers in control (DMSO) and *Pf*NDC80-deficient segmented schizonts (cKO/KD, rapamycin and glucosamine) based on expansion microscopy data (n = 24 cells). Statistical differences were determined using an unpaired Welch's t-test (**c**, ns = 0.1758 and **d**, ***p = 0.0002). **e** Ratio of the number of merozoites to the number of nuclei per cell in control and *Pf*NDC80-deficient segmented schizonts (n = 24 cells). Differences were determined using an unpaired Welch's t-test, ****p < 0.0001. **f** Quantification of the volumes of the nuclei in control (n = 452 nuclei in 20 cells) and cKO/KD (n = 545 nuclei in 20 cells) segmented schizonts. Volumes were assessed using Imaris software. The difference was determined by an unpaired t-test with Welch's correction, ****p < 0.0001. The confidence intervals of all the quantitative estimates are 95%. Supplementary Fig. 12 c, d, e, f images for analysis were acquired from three different experiments.

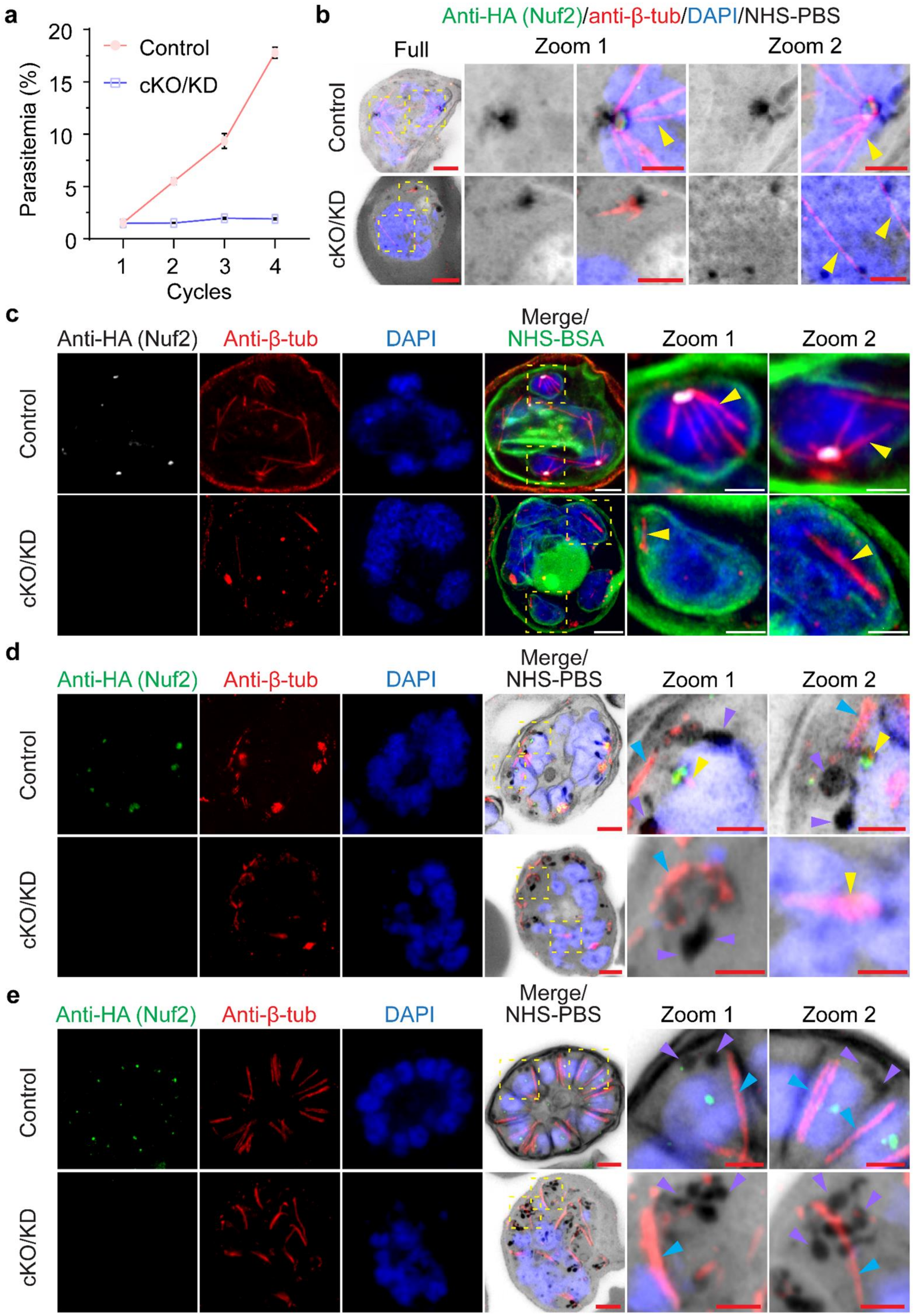

Supplementary Figure 13. *Pf*Nuf2 conditional knockout/knockdown causes killing and disrupts microtubule organization in asexual blood stage *P. falciparum*. a Asexual growth assay of *Pf*Nuf2-HA parasites. Parasites were treated with rapamycin and glucosamine (conditional knockout/knockdown, cKO/KD) or DMSO (control) and the parasitemia was monitored for 4 cycles. The experiment was repeated

3 times, and the mean and standard deviation values are shown. **b-e** U-ExM images of control and *Pf*Nuf2-cKO/KD parasites during early (**b**), mid (**c**) and later (**d**) rounds of nuclear division and in a segmented schizont (**e**). NHS-BSA (**c**, green) or NHS-PBS (**b**, **d, e** inverse greyscale) labels general proteins and DAPI (blue) labels chromatins. Anti-HA (**b**, **d, e** green or **c**, greyscale) labels the *Pf*Nuf2-HA. Anti-β-tubulin (anti-β-tub, red) marks both spindle microtubules (yellow arrowheads) and sub-pellicular microtubules (aqua arrowheads). The rhoptries are evident as NHS-PBS-labelled areas (purple arrowheads; **c** and **d**). The data relate to Fig. 5a, b. The experiment was performed three times. All images are displayed as z-projections. All scale bars are 5 μm, except the zoom images, which are 2 μm.

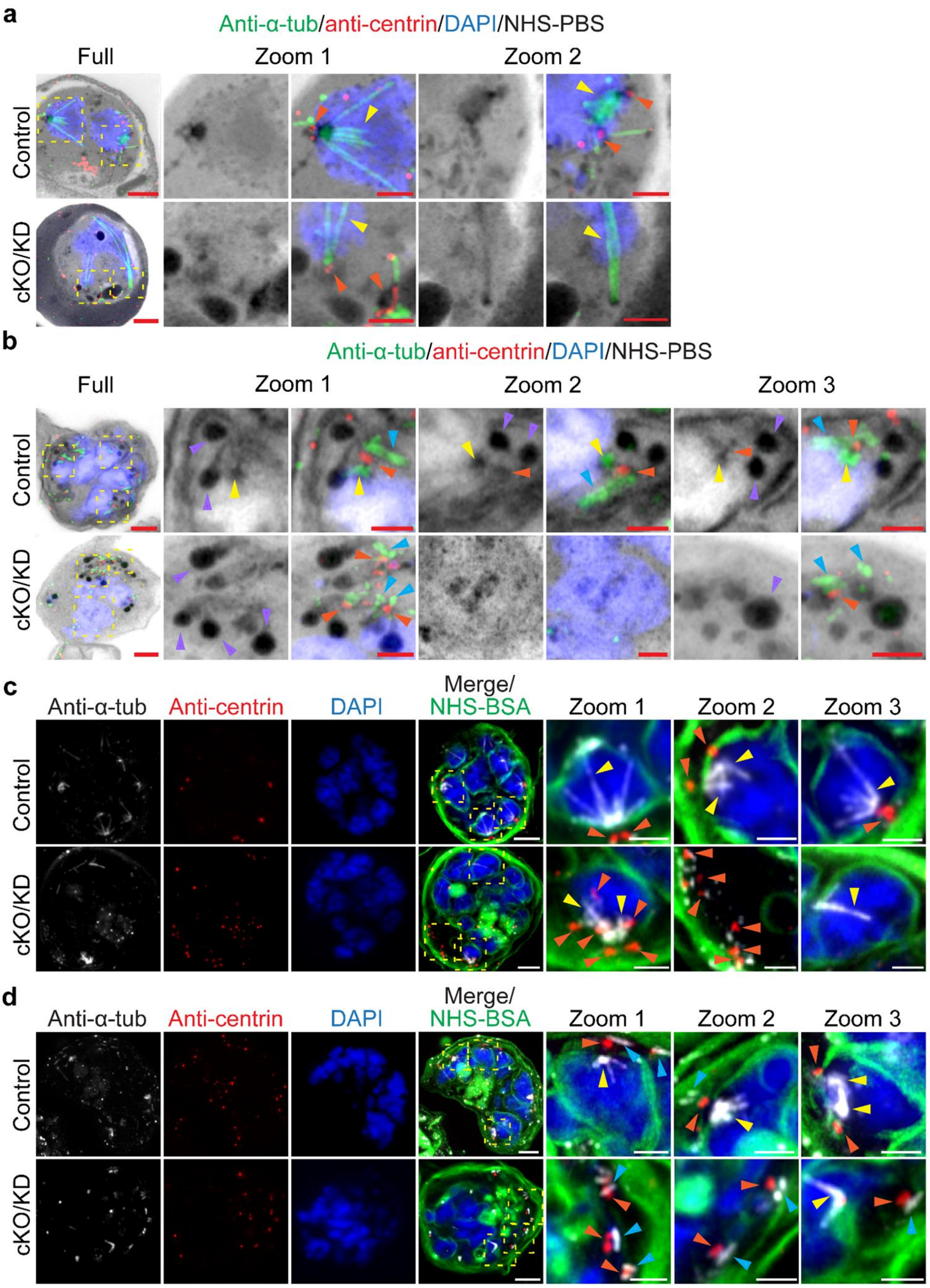

Supplementary Figure 14. *Pf*Nuf2 conditional knockout/knockdown disrupts the centrin distribution. U-ExM images of control (DMSO) and *Pf*Nuf2-deficient (conditional knockout/knockdown, cKO/KD) in early (**a**)**,** mid (**c**) and later (**b**, **d**) rounds of nuclear division. NHS-PBS (**a**, **b**, inverse greyscale) or NHS-BSA (**c**, **d**, green) are general protein dyes and DAPI (blue) labels the chromatin. The outer centriolar plaque is labelled with the anti-centrin (red, orange arrowheads) and the microtubules are labelled with anti-α-tubulin (**a**, **b**, green; **c**, **d**, greyscale, anti-α-tub). Yellow arrowheads indicate spindle microtubules and aqua arrowheads indicate sub-pellicular microtubules. Purple arrowheads show the location of rhoptries (**b**, NHS-PBS-labelled areas outside the nucleus). The data relate to Fig. 5d, e. The experiment was performed three times. All images are displayed as z-projections. All scale bars are 5 μm, except the zoom-in images which are 2 μm.

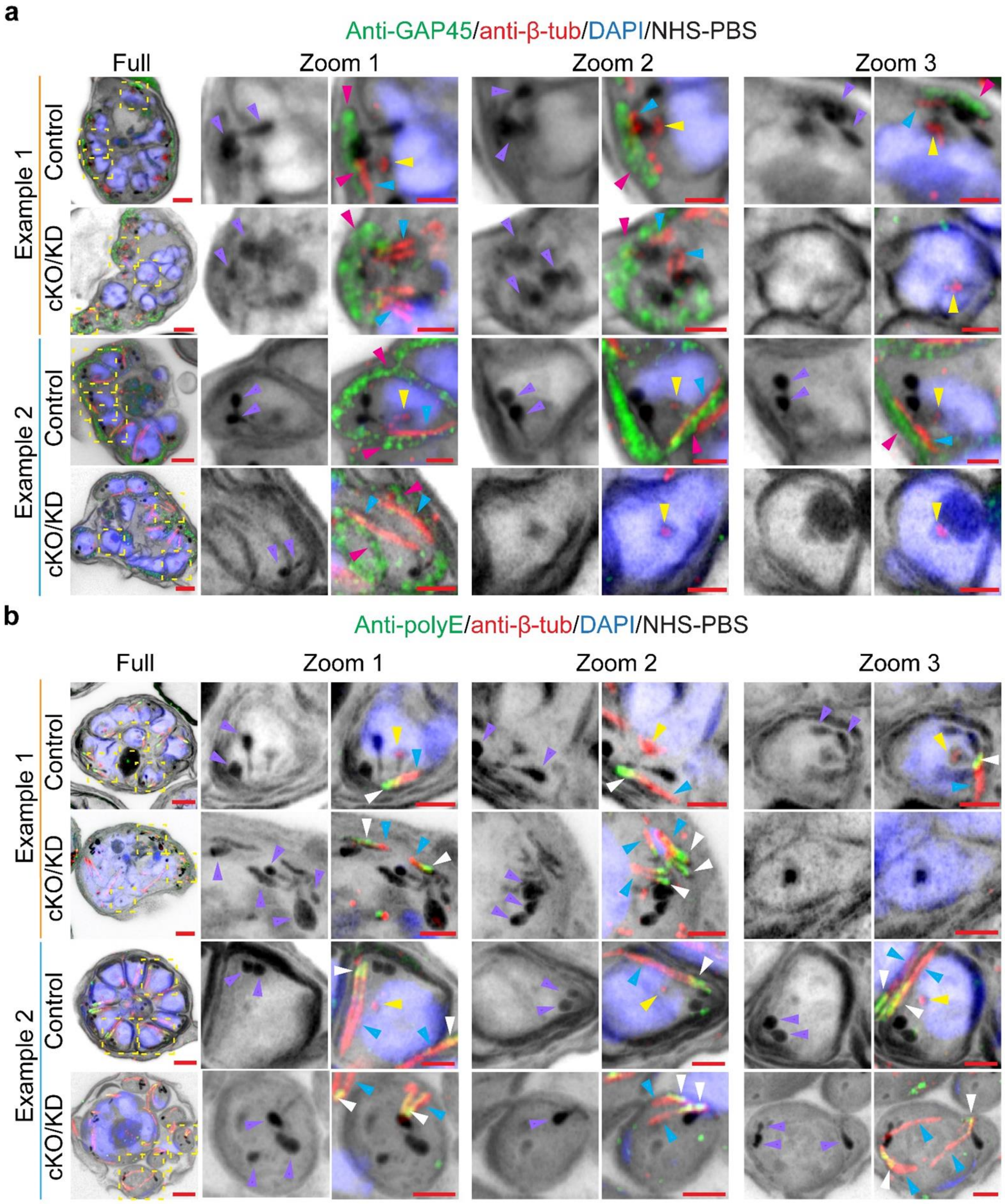

Supplementary Figure 15. *Pf*Nuf2 conditional knockout/knockdown affects the organisation of the inner membrane complex and the subpellicular microtubules in segmented schizonts. Additional U-ExM images of control and *Pf*Nuf2-deficient (conditional knockout/knockdown, cKO/KD) early segmented schizonts (**a**, **b,** Example 1) and full segmented schizonts (**a**, **b**, Example 2). NHS-PBS (inverse greyscale) and DAPI (blue). The IMC was labelled with anti-*Pf*GAP45 (**a**, green, magenta arrowheads, anti-GAP45) and polyglutamated subpellicular microtubules (aqua arrowheads) were labelled with anti-polyE (**b**, green, white arrowheads). Microtubules were labelled with anti-β-tubulin (red, anti-β-tub). The spindle microtubule remnants (yellow arrowheads) and rhoptries (purple arrowheads) are indicated. The data relate to Fig. 5f, g. The experiment was performed two times. All images are displayed as z-projections. All scale bars are 5 μm, except the zoom images, which are 2 μm.

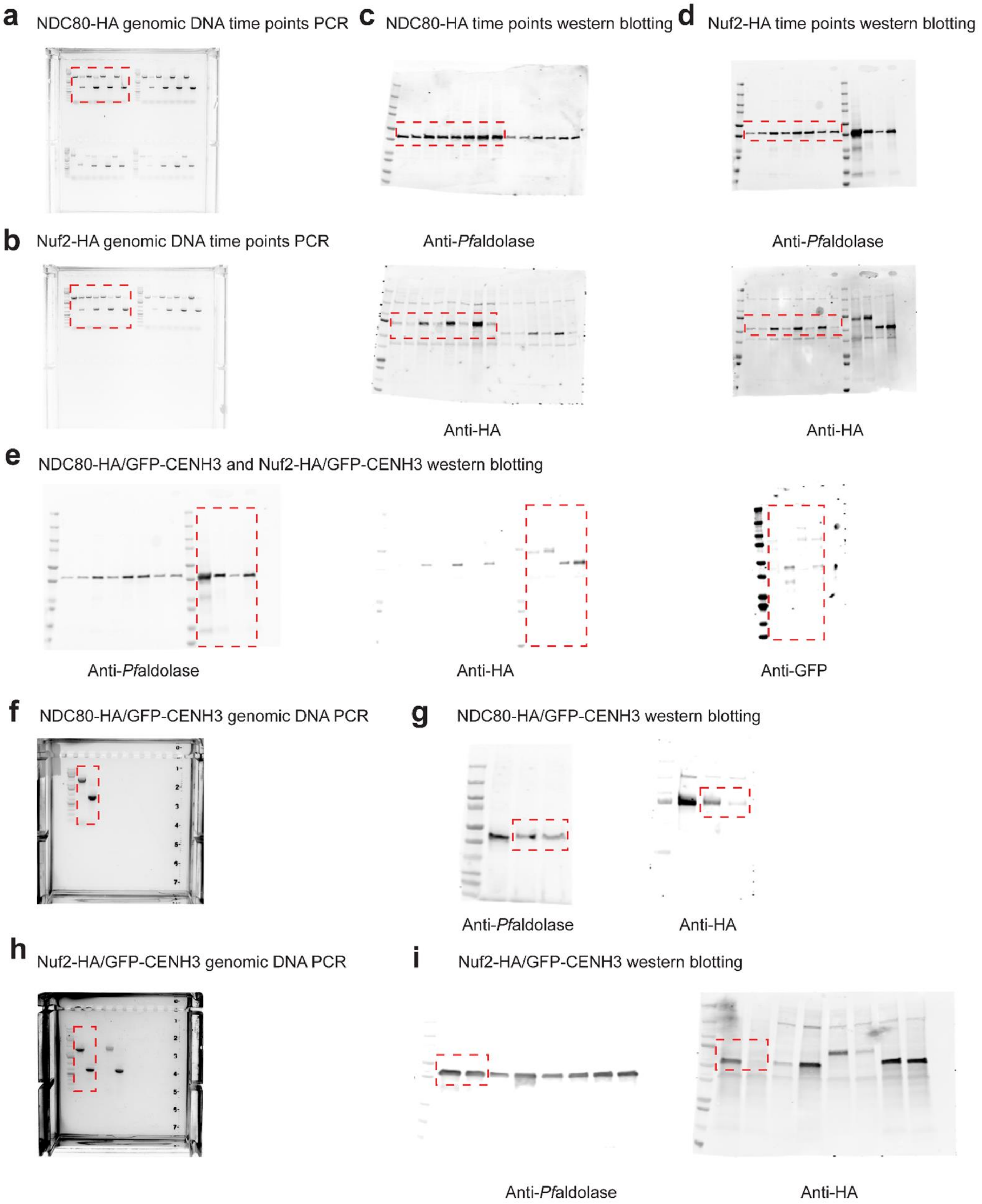

Supplementary Figure 16. Full length DNA gels and Western blots. a *Pf*NDC80-HA PCR from Supplementary Fig. 1b. **b** *Pf*Nuf2-HA PCR from Supplementary Fig. 1c. **c** Western blotting of *Pf*NDC80-HA from Supplementary Fig. 1d. **d** *Pf*Nuf2-HA Western blotting from Supplementary Fig. 1e. **e** *Pf*NDC80-HA vs *Pf*NDC80-HA/GFP-*Pf*CENH3 and *Pf*Nuf2-HA vs *Pf*Nuf2-HA/GFP-*Pf*CENH3 Western blotting from Supplementary Fig. 4b. **f** *Pf*NDC80-HA/GFP-*Pf*CENH3 PCR from Supplementary Fig. 4c. **g** *Pf*NDC80-HA/GFP-*Pf*CENH3 Western blotting from Supplementary Fig. 4d. **h** *Pf*Nuf2-HA/GFP-*Pf*CENH3 PCR from Supplementary Fig. 4f. **i** *Pf*Nuf2-HA/GFP-*Pf*CENH3 Western blotting from Supplementary Fig. 4g.

Supplementary Reference

1. Baum J*, et al.* A conserved molecular motor drives cell invasion and gliding motility across malaria life cycle stages and other apicomplexan parasites. *J Biol Chem* **281**, 5197-5208 (2006).
