# Supplementary material for: Disruption of Plasmodium falciparum kinetochore proteins destabilises the nexus between the centrosome equivalent and the mitotic apparatus
Source: Nat Commun. 2024 Jul 10;15:5794. doi: 10.1038/s41467-024-50167-6 (PMC11237077; doi:10.1038/s41467-024-50167-6)
Supplement: Supplementary file 3 — Description of Additional Supplementary Files [file 41467_2024_50167_MOESM3_ESM.docx]

**Supplementary Video legends**

**Video 1.**

**Title: 3-D array tomography reconstruction of a *Pf*NDC80-HA control (treated with DMSO) segmented schizont.**

Description: The video shows a translation through virtual sections and rendered surfaces. Colour legend: green, nucleated daughter merozoite plasma membrane; blue, nuclei; yellow, rhoptries.

**Video 2.**

**Title: 3-D array tomography reconstruction of a segmented schizont in a *Pf*NDC80-HA cKO/KD (treated with rapamycin and glucosamine) parasite.**

Description: The video shows a translation through virtual sections and rendered surfaces. Colour legend: green, nucleated daughter merozoite plasma membrane; red, anucleate daughter merozoite plasma membrane; blue, nuclei; yellow, rhoptries.
